# P-634. Settling the Score: An Analysis of Community Acquired Pneumonia Pseudomonas Risk Factor Models

**DOI:** 10.1093/ofid/ofaf695.847

**Published:** 2026-01-11

**Authors:** Joel Kennedy, Erin Weslander, Christie M Bertram, William Justin Moore, Richard G Wunderink, Sarah Sutton, Michael Postelnick, Chiagozie Ifeoma Pickens, Nathaniel J Rhodes

**Affiliations:** Northwestern Memorial Hospital, Chicago, Illinois; Northwestern Memorial Hospital, Chicago, Illinois; Northwestern Memorial Hospital/Rosalind Franklin University of Medicine and Science, Chicago, Illinois; Northwestern Medicine, Chicago, Illinois; Northwestern University Feinberg School of Medicine, Chicago, IL; Northwestern University, Chicago, Illinois; Northwestern Medicine, Chicago, Illinois; Northwestern University, Chicago, Illinois; Midwestern University, Downers Grove, IL

## Abstract

**Background:**

We previously evaluated the risk of MRSA in CAP—a rare but important phenomenon.^1^ Here, we evaluate the performance of drug-resistant Gram-negative prediction models as applied to *Pseudomonas aeruginosa* (PsA), an equally rare event, in patients with CAP to understand the strengths and weaknesses of these models.

**Table 1:**
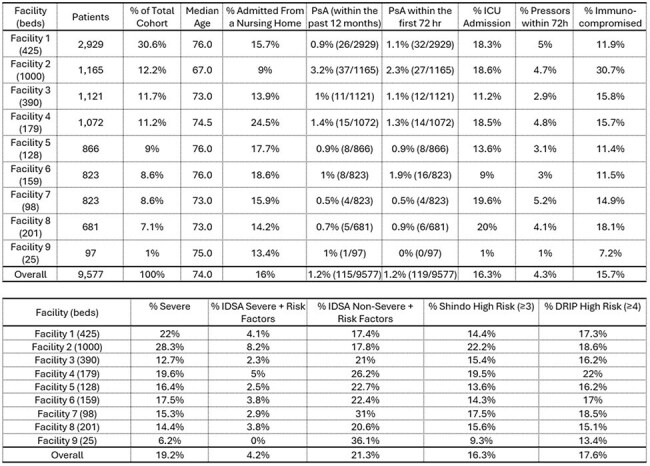
Demographics and Patient PsA Risk Categories

**Figure 1:**
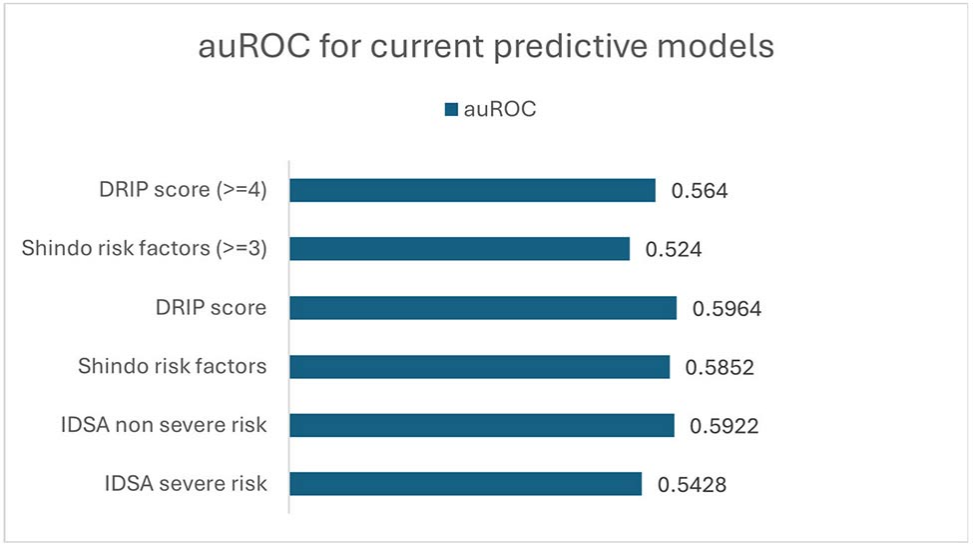
area under the Receiver Operator Curve (auROC) for Model Prediction

**Methods:**

This retrospective, IRB-approved, cohort study evaluated CAP patients admitted across NM Health System (10/1/22 to 9/30/24). De-identified patient demographics and microbiology were extracted. Non-ventilated adult (≥18 years) patients receiving CAP-directed therapy with and without septic shock were included; patients with cystic fibrosis, bronchiectasis, or who died within 48 hours of admission were excluded. Classification of PsA risk was made using established and data-driven thresholds using composite scores for a modified DRIP^2^ model (i.e., DRIP score ≥4) and a modified Shindo^3^ model (i.e., ≥3 risk factors) as well as the IDSA pathway. Univariate classification analyses were conducted using the Optimal Data Analysis package for R to generate area under the ROC (auROC) values. Confusion matrices and prediction rules were extracted after training and after leave-one-out (LOO) cross-validation.

**Figure 2:**
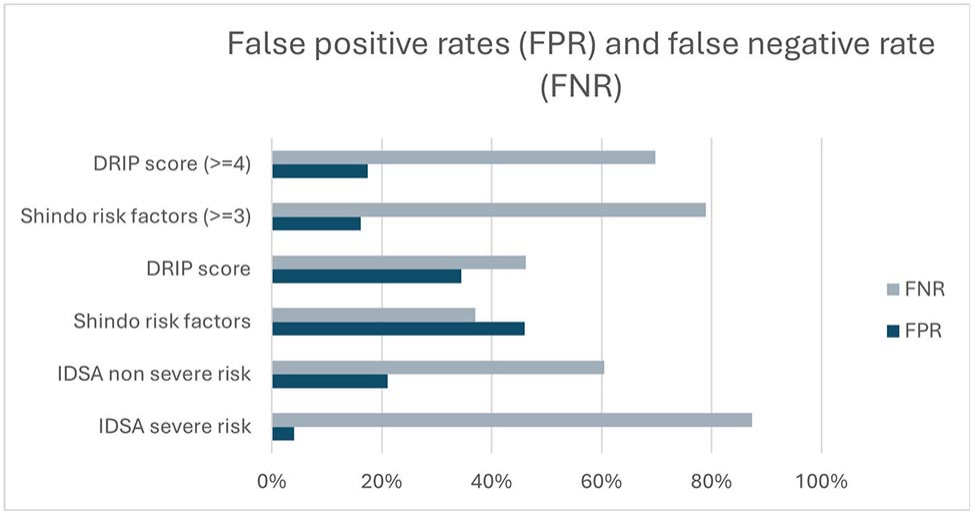
False Predictive Rate (FPR) and True Predictive Rate (TPR) for CAP PSA Prediction Models

References

**Figure:**
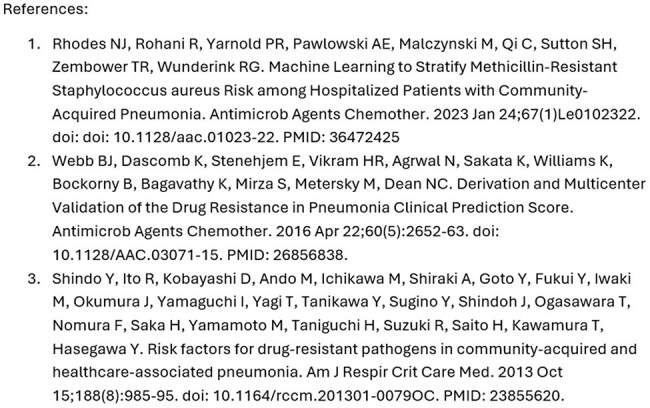

**Results:**

A total of 9,577 patients across 9 hospitals met inclusion criteria of whom 119 (1.2%) had PsA identified during their index CAP admission. Demographics varied across hospitals (Table 1). Shindo and Webb models were nondiscriminatory with auROC values 0.524 and 0.564 at established thresholds, which improved only slightly to 0.585 and 0.596 when optimal threshold values were used (i.e. ≥ 1 risk factor and DRIP score ≥ 2, respectively, Figure 1). Similarly, the IDSA classifications of severe and non-severe risk were only modestly predictive (auROC = 0.543 and 0.592, respectively). Evaluated models yielded false positive rates of 4-46% and false negative rates of 37-87% (Figure 2).

**Conclusion:**

Existing models for evaluating the risk of drug-resistant pathogens as applied to PsA in patients hospitalized with CAP are insufficiently predictive, resulting in over-calling the risk of PsA. Established scores are likely to result in substantial overuse of broad-spectrum antibiotics.

**Disclosures:**

Nathaniel J. Rhodes, PharmD MS, Apothecademy, LLC: Advisor/Consultant

